# Resveratrol Derivative-Rich Melinjo Seed Extract Attenuates Skin Atrophy in *Sod1*-Deficient Mice

**DOI:** 10.1155/2015/391075

**Published:** 2015-06-09

**Authors:** Kenji Watanabe, Shuichi Shibuya, Yusuke Ozawa, Naotaka Izuo, Takahiko Shimizu

**Affiliations:** Department of Advanced Aging Medicine, Chiba University Graduate School of Medicine, Chiba 260-8670, Japan

## Abstract

The oxidative damages induced by a redox imbalance cause age-related changes in cells and tissues. Superoxide dismutase (SOD) enzymes play a pivotal role in the antioxidant system and they also catalyze superoxide radicals. Since the loss of cytoplasmic SOD (SOD1) resulted in aging-like phenotypes in several types of murine tissue, SOD1 is essential for the maintenance of tissue homeostasis. Melinjo (*Gnetum gnemon* Linn) seed extract (MSE) contains trans-resveratrol (RSV) and resveratrol derivatives, including gnetin C, gnemonoside A, and gnemonoside D. MSE intake also exerts no adverse events in human study. In the present studies, we investigated protective effects of MSE on age-related skin pathologies in mice. Orally MSE and RSV treatment reversed the skin thinning associated with increased oxidative damage in the *Sod1* ^−/−^ mice. Furthermore, MSE and RSV normalized gene expression of *Col1a1* and *p53* and upregulated gene expression of *Sirt1* in skin tissues. *In vitro* experiments revealed that RSV significantly promoted the viability of *Sod1* ^−/−^ fibroblasts. These finding demonstrated that RSV in MSE stably suppressed an intrinsic superoxide generation *in vivo* and *in vitro* leading to protecting skin damages. RSV derivative-rich MSE may be a powerful food of treatment for age-related skin diseases caused by oxidative damages.

## 1. Introduction

Intrinsic skin aging induced by chronological or intrinsic factors leads to skin atrophy [1]. Skin collagen components show age-dependent reductions in both male and female subjects, resulting in age-related skin thinning in older individuals [2]. Accumulated evidence suggests that oxidatively modified proteins, DNA, and lipids in the skin and other organs during aging are progressively accumulated [3], indicating that reactive oxygen species (ROS) are strongly associated with skin aging. To attenuate oxidative damages, multiple antioxidative and repair systems exert in cells. Superoxide dismutase (SOD) plays a central role in the antioxidative systems due to its ability to catalyze cellular superoxide radicals (O_2_^•−^) to H_2_O_2_. H_2_O_2_ is further degraded to O_2_ and H_2_O by catalase, glutathione peroxidases, and peroxiredoxins. Copper/zinc superoxide dismutase (SOD1) is localized to react intracellular O_2_^•−^ in the cytoplasm. Our previous studies demonstrated that* Sod1*-deficient (*Sod1* ^−/−^) mice showed enhancement of intracellular O_2_^•−^ and various aging-like organ phenotypes, suggesting that cytoplasmic O_2_^•−^-mediated oxidative damages primarily cause aging-like changes in various tissues [4]. Particularly,* Sod1* insufficiency resulted in both epidermal and dermal atrophies associated with downregulation of extracellular matrix-related genes including* Col1a1* and with upregulation of age-related genes including p53 [5, 6]. Therefore,* Sod1* ^−/−^ mouse is a suitable model for studying skin aging in older people.

Melinjo (Indonesian name;* Gnetum gnemon* Linn) is an arboreal dioecious plant that is widely cultivated in Southeast Asia. Its fruits and seeds are used as an ordinary vegetable in Indonesia. Melinjo seeds contain various stilbenoids including trans-resveratrol (3,5,4′-trihydroxy-trans-stilbene), its glucoside, resveratrol dimer (gnetin C), and resveratrol dimer glucoside (gnetin L, gnemonoside A, gnemonoside C, and gnemonoside D) [7]. Melinjo seed extract (MSE) revealed DPPH radical scavenging [7], lipase and *α*-amylase inhibitory [7], antimicrobial, immunostimulatory [7], angiogenesis inhibitory [8], tyrosinase inhibitory activities [9], promotion of melanin biosynthesis [9], and prevention of endothelial senescence [10]. Recently, Tatefuji et al. also reported that acute and subchronic MSE administration showed no adverse effect in rat [11]. In human study, MSE administration decreases the serum uric acid levels by inhibiting the reabsorption of uric acid in the renal tubular epithelia as well as by increasing the HDL cholesterol levels by PPAR agonistic activity with no cause of the damage to health [12]. Furthermore, Tani et al. reported that single and repeated administration of MSE demonstrated no clinical noteworthy abnormalities [13]. MSE contains 1.2 mg/g (5.26 *μ*mol/g) of RSV [13], while average content of RSV was 1.04 mg/L in red wine [14]. Therefore, MSE becomes a nutrient source of RSV with harmless long-term intake. RSV has been identified as a* Sirt1* activator that has been shown to protect various organs against aging [15, 16]. Furthermore, RSV possesses antioxidative activity and protective effect of ROS- and ultraviolet-induced cell death [17, 18]. In addition,* Sirt1* is a key modulator of cellular pathways involved in inherited dermatologic diseases and skin cancers [19], suggesting that* Sirt1* activation is a molecular target for dermatological therapy. In the present study, we investigated antiatrophic effects of MSE and RSV on age-related skin pathologies in* Sod1* ^−/−^ mice.

## 2. Materials and Methods

### 2.1. Reagents

MSE (Lot number YMP-M-110115) was provided by the Institute for Bee Products & Health Science, Yamada Bee Company, Inc. (Okayama, Japan). The MSE contains trans-resveratrol (RSV, 0.10% w/w), gnetin C (2.03% w/w), gnemonoside A (16.35% w/w), and gnemonoside D (3.97% w/w). Resveratrol (RSV) was obtained from Tokyo Chemical Industry Co. Ltd. (CAS 501-36-0, Tokyo, Japan). The purity of RSV is more than 98%.

### 2.2. Mice and Diets

*Sod1* ^−/−^ mice were purchased from the Jackson Laboratory (Bar Hrbor, ME, USA). The genotyping of* Sod1* ^−/−^ allele was performed using genomic PCR with genomic DNA isolated from the tail tip, as previously reported [20]. The animals were housed in a room temperature of 24 ± 1°C, a relative humidity of 55 ± 10%, and a 12 h light/dark cycle and were fed* ad libitum*. Experimental procedures were approved by the Animal Care and Use Committee of Chiba University. At 4 weeks of age, mice were randomly divided into four groups and fed respective experimental diets for 12 weeks: control MF diet (composition: 7.9% water, 21.1% protein, 5.1% lipid, 5.8% ash, 2.8% fiber, and 55.3% soluble nitrogen free extract, 359 kcal/100 g, Oriental Yeast Co., Ltd., Tokyo, Japan), control MF diet containing 0.04% (w/w) RSV as previously described [15], and control MF diets containing 0.1% or 0.5% (w/w) MSE (Lot number YMP-M-110115) according to the previous study [8].

### 2.3. Histology

For histological morphology, skin specimens from back tissues were dissected and fixed in a 20% formalin neutral buffer solution (Wako, Osaka, Japan) overnight. After dehydration and penetration, skin specimens were embedded in paraffin and sectioned on a microtome (ROM-380, Yamato Koki Kogyo Co. Ltd., Saitama, Japan) at 4 *μ*m thickness by standard techniques. Hematoxylin and eosin staining for skin morphology and Sirius red staining for total collagen deposition were performed as described previously [21–23]. The thickness of the skin tissue was measured using Leica QWin V3 image software (Leica, Germany).

### 2.4. Measurement of Oxidative Stress Markers

In order to measure the 8-isoprostane content, blood was collected from the left ventricular space and centrifuged at 12,000 rpm for 5 min at room temperature. Plasma was separated from the clotted blood and added 100 *μ*M indomethacin and 0.005% dibutylhydroxytoluene. The 8-isoprostane level was measured using the 8-isoprostane EIA Kit (Cayman Chemical Company) according to the manufacturer's instructions. The plasma was also assayed for the protein concentration using the DC Protein Assay Kit (Bio-Rad, Hercules, CA, USA), and 8-isoprostane levels were normalized to the protein level.

For intracellular ROS measurement, bone marrow cells (5–10 × 10^5^ cells/two tibias of a mouse) were collected by flushing tibias with phosphate-buffered saline using 26-G needles and stained with 10 *μ*M of CM-H_2_DCFDA (DCF, Life Technologies Corporation) for 30 min at 37°C. Primary dermal fibroblasts were incubated with 10 *μ*M DCF for 30 min at 37°C. After incubation, cells were trypsinized and resuspended in PBS. Their fluorescence intensities were assessed using a flow cytometer (BD FACSCanto II, BD Biosciences).

### 2.5. Cell Culture

Skin tissues were dissected from* Sod1* ^−/−^ neonates at 5 days of age. The primary dermal fibroblasts were isolated by dissociation in 0.2% collagenase type 2 (Worthington Biochemical Corporation Lakewood, NJ, USA) at 37°C for 60 min. Cells were cultured in *α*-MEM (Life Technologies Corporation, Carlsbad, CA, USA) supplemented with 20% fetal bovine serum (FBS), 100 unit/mL penicillin, and 0.1 mg/mL streptomycin at 37°C in a humidified incubator with 5% CO_2_ and 1% O_2_. Cells were treated with 10 *μ*M RSV at 72 h. We determined the concentration and duration of RSV treatment in this study according to our previous paper [6].

### 2.6. Outgrowth Assay

The back skin was sterilized with 70% ethanol, rinsed with PBS (Takara Bio Inc., Shiga, Japan), and punched out into discs measuring 5 mm in diameter using dermal punch (Nipro, Tokyo, Japan). The punched skin discs were placed into a 12-well culture plate (Falcon BD, Franklin Lakes, NJ) and cultured with or without 10 *μ*M RSV in *α*-MEM containing 20% FBS, 100 units/mL of penicillin, and 0.1 mg/mL of streptomycin at 37°C in a humidified incubator with 5% CO_2_ and 1% O_2_. The number of outgrowth fibroblasts originating from the mouse skin disc was directly counted at 72 h after culture. The method of this experiment was performed as described previously [5].

### 2.7. Quantitative PCR

Total RNA was extracted from back skin using the Trizol reagent (Life Technologies Corporation, Carlsbad, CA, USA) according to the manufacturer's instructions. cDNA was synthesized from 1 *μ*g of total RNA using reverse transcriptase (ReverTra Ace qPCR RT Master Mix, Toyobo, Osaka, Japan). Real-time PCR was performed on a MiniOpticon (Bio-Rad) with the SYBR Green PCR Master Mix (Bio-Rad) according to the manufacturer's instructions. All data were normalized to the level of the housekeeping gene glyceraldehyde-3-phosphate dehydrogenase (*Gapdh*). The following primers were used for the analysis:* Gapdh*, forward, 5′-ATGTGTCCGTCGTGGATCTGA-3′, and reverse, 5′-TGCCTGCTTCACCACCTTCT-3′;* Col1a1*, forward, 5′-CATGTTCAGCTTTGTGGACCT-3′, and reverse, 5′-GCAGCTGACTTCAGGGATGT-3′;* p53*, forward, 5′-ACGCTTCTCCGAAGACTGG-3′, and reverse, 5′-AGGGAGCTCGAGGCTGATA-3′;* Sirt1*, forward, 5′-CAGTGAGAAAATGCTGGCCTA-3′, and reverse, 5′-TTGGTGGTACAAACAGGTATTGA-3′.

### 2.8. Statics

The statistical analyses were performed using Student's* t*-test for comparisons between two groups and Tukey's test for comparisons among three groups. Differences between the data were considered significant when the *P* values were less than 0.05. All data are expressed as the mean ± standard deviation (SD).

## 3. Results

### 3.1. MSE and RSV Attenuate the Skin Atrophy of* Sod1* ^−/−^ Mice

SOD1, one of the cellular antioxidant enzymes, plays a pivotal role in regulating oxidative and reductive balance.* Sod1* ^−/−^ mice showed age-related atrophic morphology in their skin accompanied by the degeneration of collagen [5]. Therefore, we have used* Sod1* ^−/−^ mice for skin aging research and for screening of antiatrophic compounds in skin thickness [24–26]. In this context, we investigated antiatrophic effects of MSE and RSV on the skin thickness of* Sod1* ^−/−^ mice.

**Figure 1:**
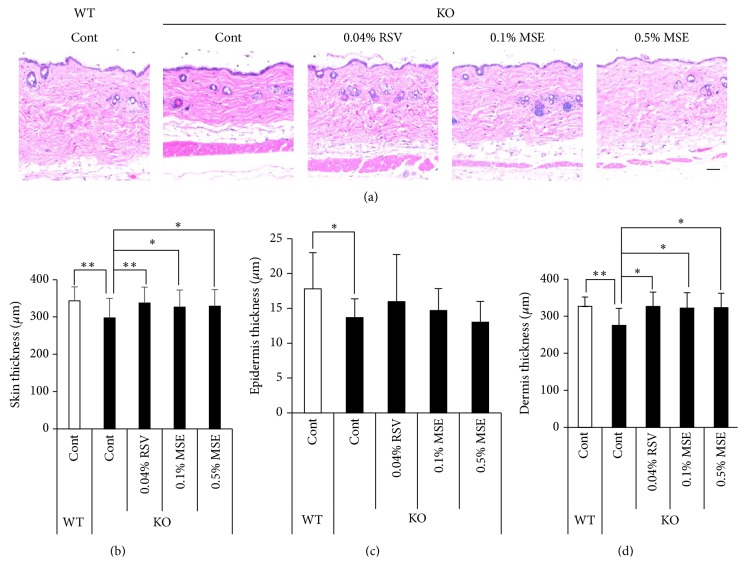
MSE and RSV attenuate skin atrophy in the* Sod1* ^−/−^ mice. (a) Hematoxylin and eosin staining of the back skin of* Sod1* ^−/−^ (KO) and* Sod1* ^+/+^ (WT) mice treated with the MSE or RSV. MSE and RSV containing diets were administrated for 12 weeks. The thickness of (b) total, (c) epidermis, and (d) dermis of the back skin of the* Sod1* ^−/−^ and* Sod1* ^+/+^ mice treated with MSE or RSV (*n* = 10–12). The statistical evaluations were performed using the Tukey's test. These data indicate the mean ± SD; ^*∗*^ *P* < 0.05, ^*∗∗*^ *P* < 0.01. The scale bar represents 100 *μ*m.

**Figure 2:**
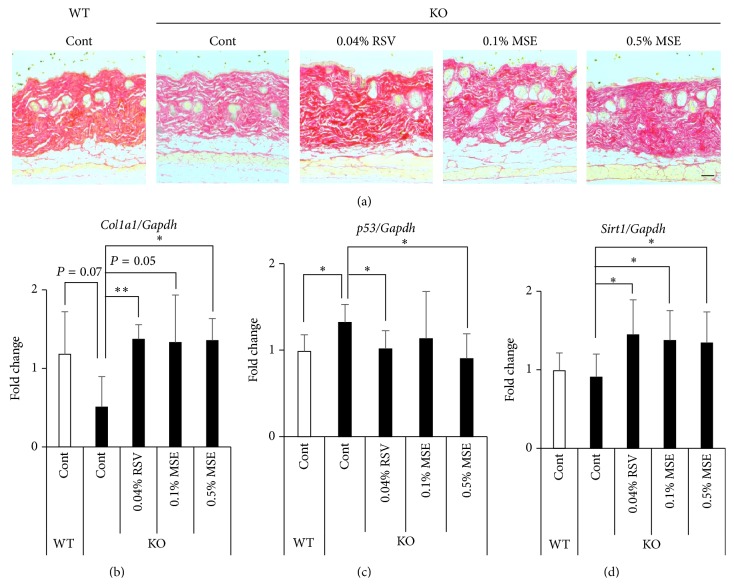
MSE and RSV attenuate collagen decline in skin tissues of* Sod1* ^−/−^ mice. (a) Sirius red staining of the back skin of* Sod1* ^−/−^ and* Sod1* ^+/+^ mice treated with the MSE or RSV. Relative mRNA expression of (b)* Sirt1*, (c)* Col1a1*, and (d)* p53*. Each of the mRNA expressions was determined by qRT-PCR (*n* = 8–12). The statistical evaluations were performed using the two-tailed Student's* t*-test for unpaired values. These data indicate the mean ± SD; ^*∗*^ *P* < 0.05, ^*∗∗*^ *P* < 0.01. The scale bar represents 100 *μ*m.

In a preliminary experiment, MF diets containing 5% or 0.5% MSE were orally administrated to the* Sod1* ^+/+^ and* Sod1* ^−/−^ mice daily for 12 weeks beginning at 4 weeks of age. The results showed that both MSE diets improved the skin thickness of* Sod1* ^−/−^ mice and there were no adverse effects of skin pathologies of* Sod1* ^+/+^ mice (data not shown). Therefore, we selected the control diet containing 0.5% MSE to confirm antiatrophic effect on* Sod1* ^−/−^ skin. MSE and RSV were orally administered to the* Sod1* ^+/+^ and* Sod1* ^−/−^ mice under the same conditions. As shown in Figure 1(a), the skin of* Sod1* ^−/−^ mice was significantly thinner compared to that of* Sod1* ^+/+^ mice, confirming skin atrophy in* Sod1* ^−/−^ mice. The back skin of* Sod1* ^−/−^ that had been administrated with the MSE diets was significantly thicker compared to that of* Sod1* ^−/−^ mice treated with the control diet (Figures 1(b)–1(d)). RSV diet also improved skin atrophy of* Sod1* ^−/−^ mice compared to* Sod1* ^−/−^ mice treated with the control diet (Figures 1(b)–1(d)). To investigate the adverse effect of MSE and RSV diets, we similarly administered MSE and RSV to the* Sod1* ^+/+^ mice. No significant difference in skin thickness and morphology was observed in* Sod1* ^+/+^ mice treated with MSE and RSV (data not shown), indicating that MSE and RSV were safety food factors in skin during short-time treatment. In addition, Sirius red staining revealed that the skin of* Sod1* ^−/−^ mice was decreased in staining intensity compared to that observed in* Sod1* ^+/+^ mice (Figure 2(a)), confirming dermal collagen decline in* Sod1* ^−/−^ mice. Notably, both MSE and RSV diets increased the Sirius red intensity in* Sod1* ^−/−^ dermis (Figure 2(a)), implying enhancement of collagen level in* Sod1* ^−/−^ skin.

### 3.2. MSE and RSV Alter Gene Expression in* Sod1* ^−/−^ Skin

To investigate skin atrophy-preventing mechanism of MSE and RSV on skin atrophy in* Sod1* ^−/−^, we analyzed expression patterns of type I collagen and age-related genes in skin. In* Sod1* ^−/−^ skin, mRNA level of* Col1a1* was significantly downregulated compared to those of* Sod1* ^+/+^, indicating reduced collagen biosynthesis (Figure 2(b)). Moreover, p53, one of the major age-related genes, also significantly upregulated in* Sod1* ^−/−^ skin (Figure 2(c)). MSE and RSV treatment significantly normalized mRNA levels of* Col1a1* and* p53* in* Sod1* ^−/−^ skin (Figures 2(b) and 2(c)). Interestingly, we revealed that MSE and RSV treatment also significantly upregulated* Sirt1* expression, suggesting the molecular link between* Sirt1* expression and skin thinning in* Sod1* ^−/−^ mice (Figure 2(d)). These findings demonstrated that application of MSE and RSV diets improved the skin atrophy accompanied by normalization and activation of age-related genes in* Sod1* ^−/−^ mice.

### 3.3. MSE and RSV Significantly Attenuate Oxidative Damage in* Sod1* ^−/−^ Mice

**Figure 3:**
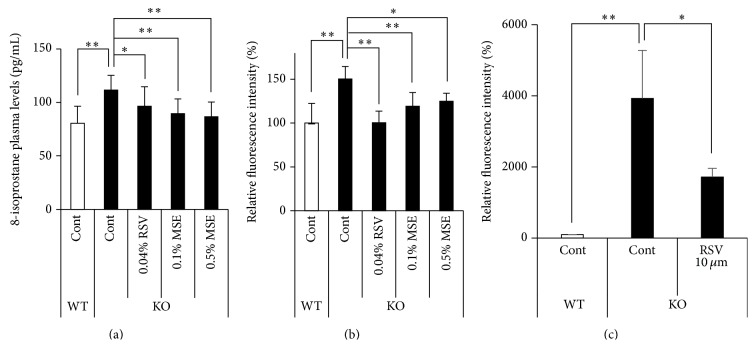
MSE and RSV decrease oxidative damage and ROS production. (a) 8-isoprostane content in plasma obtained from* Sod1* ^−/−^ and* Sod1* ^+/+^ mice treated with MSE and RSV (*n* = 10–12). (b) The intracellular ROS levels of bone marrow cells of* Sod1* ^−/−^ and* Sod1* ^+/+^ mice were measured using a DCF dye (*n* = 5-6). (c) The relative intracellular ROS level in* Sod1* ^−/−^ fibroblasts treated with 10 *μ*M RSV for 72 h was measured by a DCF dye (*n* = 3). The statistical evaluations were performed using the Tukey's test. These data indicate the mean ± SD; ^*∗*^ *P* < 0.05, ^*∗∗*^ *P* < 0.01.

*Sod1* ^−/−^ mice showed significant increase of several oxidative damage markers, including lipid peroxidation, in tissues [20, 24, 27, 28]. In order to evaluate oxidative damage, we measured the lipid peroxidation levels in the plasma. Regarding the 8-isoprostane levels, MSE and RSV containing diets significantly reduced the 8-isoprostane content in the plasma (Figure 3(a)). Furthermore, MSE and RSV containing diets decreased intracellular ROS level in cells from bone marrow (Figure 3(b)). These data indicate that MSE and RSV treatment mitigated oxidative damage in* Sod1* ^−/−^ mice.

### 3.4. MSE and RSV Significantly Restore Viability in* Sod1* ^−/−^ Fibroblasts

**Figure 4:**
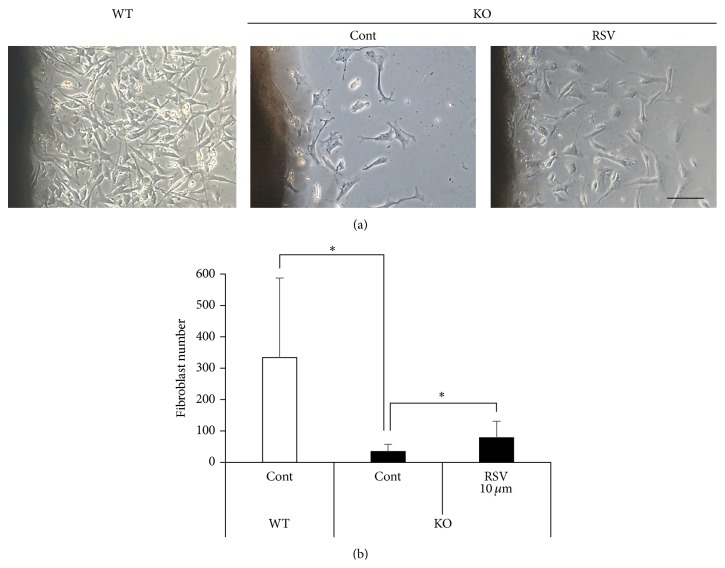
RSV promotes fibroblasts outgrowth from* Sod1* ^−/−^ skin. (a and b) Number of outgrowth fibroblasts of* Sod1* ^+/+^ and* Sod1* ^−/−^ mice in the skin disc culture treated with 10 *μ*M RSV for 72 h (*n* = 8). Fibroblast number was counted on day 3. The statistical evaluations were performed using the two-tailed Student's* t*-test for unpaired values. These data indicate the mean ± SD; ^*∗*^ *P* < 0.05. The scale bar represents 100 *μ*M.

We investigated whether the RSV treatment attenuated intracellular ROS production and promoted the proliferation of* Sod1* ^−/−^ fibroblasts* in vitro*. Preliminary experiments revealed that RSV treatment for 24 h with various concentrations of 30 to 100 *μ*M slightly suppressed cell viability of* Sod1* ^+/+^ fibroblasts. In contrast, 10 *μ*M RSV treatment for 72 h showed no adverse effect of cell viability in* Sod1* ^+/+^ fibroblasts. Therefore, we determined dose and duration of the RSV experiment* in vitro*. Flow cytometer analysis indicated that RSV treatment significantly decreased intracellular ROS generation in* Sod1* ^−/−^ fibroblasts (Figure 3(c)). Moreover, the organ culture experiments using skin discs revealed that the* Sod1* ^−/−^ fibroblasts showed marked suppression of their outgrowth capacity compared to that observed in the* Sod1* ^+/+^ mice (Figure 4(a)). Treatment with 10 *μ*M RSV significantly enhanced the fibroblasts outgrowth activity from the* Sod1* ^−/−^ skin discs (Figure 4(b)). These findings collectively suggested that the RSV promoted the migration and proliferation of* Sod1* ^−/−^ fibroblasts* in vitro*.

## 4. Discussion

In the present study, we demonstrated that MSE and RSV significantly reversed skin thinning via reduction of oxidative damages in* Sod1* ^−/−^ mice (Figure 1). Recently, we have reported that* Sod1* ^−/−^ fibroblasts showed excessive ROS accumulation associated with mitochondrial dysfunction [6].* In vitro* study also revealed that RSV treatment significantly reduced intracellular ROS generation and restored cell viability in* Sod1* ^−/−^ fibroblasts (Figures 3(c) and 4). Accumulating evidence revealed that RSV activates mitochondrial function and antioxidant defense leading to suppressing ROS generation [29]. Furthermore, SIRT1 also increases mitochondrial function and biogenesis and promotes cell proliferation and migration [15, 30, 31]. In a human study, treatment with a nutraceutical supplement containing resveratrol, procyanidin, and ellagic acid induced reduction of skin wrinkling, as well as reducing systemic and skin oxidative stress in a clinical setting [32]. These findings suggested that the* Sirt1*-mediated antioxidant activities of RSV contribute to attenuate skin damages in mammals. To rescue age-related changes in tissues of* Sod1* ^−/−^ mice, we have evaluated beneficial effects of several antioxidants* in vivo*. Ascorbic acid administration significantly attenuated bone loss and fragility of* Sod1* ^−/−^ mice [28]. Transdermal administration of ascorbic acid derivatives also normalized skin thinning of* Sod1* ^−/−^ mice [25, 26]. Furthermore, Iuchi et al. reported that oral N-acetylcysteine treatment mitigated hemolytic anemia of* Sod1* ^−/−^ mice by suppressed ROS generation in red blood cells [27]. Recently, Shibuya et al. showed that an SOD/catalase mimetic, PAPLAL, treatment attenuated skin atrophy [33]. These reports strongly supported that antioxidants, such as RSV, ascorbic acid, N-acetylcysteine, and PAPLAL, positively improved oxidative damage-induced organ pathologies.

As shown in Figure 2(c),* Sod1* deficiency showed upregulation of* p53* gene expression, which regulates cellular senescence and death, in skin (Figure 2(c)). We previously reported that* Sod1* loss induced O_2_^•−^ generation and upregulated p53 protein level in skin fibroblasts [6]. Ascorbic acid derivatives significantly downregulated p53 expression and improved cell viability in* Sod1* ^−/−^ fibroblasts [6, 25]. In a genetically modified model, p53 activation induced accelerated aging-like phenotypes, including skin atrophy, in p53 mutant mice [34]. Gannon et al. also reported that p53 activation by* Mdm2*-specific loss in keratinocytes induced epidermal stem cell senescence and atrophy in mice, suggesting that p53 activation in skin accelerated aging-like skin thinning in mice [35]. MSE and RSV treatment significantly downregulated mRNA level of* p53* in* Sod1* ^−/−^ skin (Figure 2(c)). These data indicated that MSE and RSV treatment may delay skin aging via reducing the p53 upregulation in skin.

RSV promotes the activity and expression of* Sirt1* [15]. MSE and RSV also normalized the gene expression of* Col1a1* and upregulated the gene expression of* Sirt1* in skin of* Sod1* ^−/−^ mice (Figures 2(b) and 2(d)).* Sirt1* upregulation by RSV may protect skin aging from oxidative damage in* Sod1* ^−/−^ mice. Actually, Lee et al. reported that RSV treatment or* Sirt1* overexpression significantly inhibited matrix metalloprotease-9 expression and appeared to protect collagen from degradation after ultraviolet radiation in human dermal fibroblasts and skin tissues [36]. Serravallo et al. reported that* Sirt1* plays a pivotal role in modulating skin diseases including psoriasis, autoimmune disease, cutaneous fungal infection, inherited dermatological diseases, and cancer [19]. These findings indicated that upregulation of* Sirt1* expression protected skin damages* in vivo*.

Recently, Konno et al. reported that RSV and MSE showed the agonistic activity for PPAR*α* and PPAR*γ in vitro* [12]. It is reported that a PPAR*α*/*γ* dual agonist, MHY966, treatment significantly suppressed UVB-induced collagen digestion, lipid peroxidation, and inflammatory response via activating PPAR*α* and PPAR*γ* in mouse skin during photoaging [37]. Moreover, Mastrofrancesco et al. reported that PPAR*γ* activation in skin normalized inflammatory response in IL-21-induced epithelial hyperplasia in mice [38]. These reports suggested that RSV and MSE may activate PPAR*α* and PPAR*γ* leading to attenuating the skin atrophy in* Sod1* ^−/−^ mice.

Finally, we, here, focused on RSV in MSE and antiatrophic effects of RSV in* Sod1* ^−/−^ skin. Since MSE also contains several RSV-derivatives such as gnetin C, gnemonoside A, and gnemonoside D, we cannot rule out antiatrophic effects of the RSV derivatives in MSE. Further analysis should be needed to clarify the beneficial effect of other RSV derivatives in MSE on skin atrophy in* Sod1* ^−/−^ mice.

## 5. Conclusion

In the present study, we demonstrated that MSE and RSV treatment effectively attenuated aging-like skin pathologies accompanied by upregulation of* Sirt1* expression in* Sod1* ^−/−^ skin. MSE and RSV also exhibited less adverse effect on skin morphology. Consistent with our results, many interventions reported safety of MSE and RSV treatment in human. Therefore, MSE is useful for nutrient source of RSV as well as safety antioxidant for delaying skin aging in humans.
